# Obituary—Dr. Bradley

**Published:** 1891-01

**Authors:** 


### Obituary—Dr. Bradley.

Died at his home in Dayton, Ohio, November 1st, 1890, of cerebritis, Dr. Calvin Bradley.

He was born in Franklin, O. He went to Dayton in 1842, and began the study of dentistry under the direction of Dr. James Jones, then the leading dentist of that city. He began the practice of dentistry in 1846, having devoted two years of close application in work and study preparatory for the practice of his profession. The progress he made, and the efficiency he attained in his pupilage under so thorough a master, showed itself ever after in his professional career. For forty years he maintained an enviable reputation and position, not only in the profession, but in public estimation as well.

He had many warm friends in the profession; all who knew him admired him. He was not as widely known as his merit would warrant, owing to his retiring manner and disposition. He always avoided notoriety and would not permit himself to be placed in a prominent position. To younger members of the profession he was ever kind and obliging, and his helping hand and ready advice will long be missed, and as long remembered. His special friendships were not very numerous, but when once formed were true and neverfailing. It was the good fortune of the writer to form the acquaintance of Dr. Bradley nearly forty years ago, and in all this time has known him as the true, genial, sincere and warm-hearted friend that showed itself at first acquaintance. Dr. Bradley known, was Dr. Bradley esteemed and loved.

Though he is gone, his life and character stands as an example for all those who remain, as well as for those who shall come after.

